# *De Novo* Transcriptome Analysis of an Aerial Microalga *Trentepohlia jolithus:* Pathway Description and Gene Discovery for Carbon Fixation and Carotenoid Biosynthesis

**DOI:** 10.1371/journal.pone.0108488

**Published:** 2014-09-25

**Authors:** Qianqian Li, Jianguo Liu, Litao Zhang, Qian Liu

**Affiliations:** 1 Institute of Oceanology, Chinese Academy of Sciences, Qingdao, China; 2 University of the Chinese Academy of Sciences, Beijing, China; University of North Carolina at Charlotte, United States of America

## Abstract

**Background:**

Algae in the order Trentepohliales have a broad geographic distribution and are generally characterized by the presence of abundant β-carotene. The many monographs published to date have mainly focused on their morphology, taxonomy, phylogeny, distribution and reproduction; molecular studies of this order are still rare. High-throughput RNA sequencing (RNA-Seq) technology provides a powerful and efficient method for transcript analysis and gene discovery in *Trentepohlia jolithus*.

**Methods/Principal Findings:**

Illumina HiSeq 2000 sequencing generated 55,007,830 Illumina PE raw reads, which were assembled into 41,328 assembled unigenes. Based on NR annotation, 53.28% of the unigenes (22,018) could be assigned to gene ontology classes with 54 subcategories and 161,451 functional terms. A total of 26,217 (63.44%) assembled unigenes were mapped to 128 KEGG pathways. Furthermore, a set of 5,798 SSRs in 5,206 unigenes and 131,478 putative SNPs were identified. Moreover, the fact that all of the C4 photosynthesis genes exist in *T. jolithus* suggests a complex carbon acquisition and fixation system. Similarities and differences between *T. jolithus* and other algae in carotenoid biosynthesis are also described in depth.

**Conclusions/Significance:**

This is the first broad transcriptome survey for *T. jolithus*, increasing the amount of molecular data available for the class Ulvophyceae. As well as providing resources for functional genomics studies, the functional genes and putative pathways identified here will contribute to a better understanding of carbon fixation and fatty acid and carotenoid biosynthesis in *T. jolithus*.

## Introduction

The order Trentepohliales (Chlorophyta: Ulvophyceae) is one of the most widespread groups of organisms, and represents an ideal model taxon to investigate evolutionary patterns [1]. Species of this order are widely distributed over an extensive range of habitats in areas with humid and rainy climates including tropical and temperate regions [2]–[4]. Many of these species have been reported as lichen photobionts and to produce carbohydrates for fungi [5]–[7]. As the only family within the Trentepohliales, with six genera, the Trentepohliaceae is generally characterized by the presence of abundance β-carotene and hematochrome, which color the algal thallus yellow, orange, or red [1]. It is also characterized by the absence of pyrenoids in the chloroplast, a flagellar apparatus with unique ultrastructure, transverse cell walls with plasmodesmata, and differentiated reproductive cells [1], [3]. In recent years, numerous studies have been carried out on many aspects of the biology of *Trentepohlia*. Many of these monographs are concerned with morphology, taxonomy, phylogeny, distribution and reproduction [2], [7]–[13]. Diversity, life history and ecology were examined in detail by Rindi et al. [14]. Others have investigated physiology, photosynthesis and ultrastructure [6], [7], [15]–[18]. The classification and phylogeny of the Trentepohliales at the genus level have been determined, using morphological characteristics and molecular data [1], [3], respectively.

*Trentepohlia* Martius 1817 was first described by Printz in 1939, and afterwards detailed studies on *Trentepohlia* were carried out over a very long period of time [8], [19]. These aeroterrestrial phototrophic microorganisms typically grow epiphytically and epilithically on natural surfaces such as tree bark, soil and rock [20]. In addition, they are also found in urban areas on anthropogenic surfaces such as bricks, concrete, cement and other artificial surfaces [21]. These rock-inhabiting algae are a kind of lithophytic alga that lives on, or within, rock substrates, expanding to a few millimeters underneath the rock surface [22]. The nutrient supply of aeroterrestrial algae, however, has not been so well established [23]. Lange et al. proposed that green algal lichens are capable of photosynthesizing in the presence of water vapor [24]. Wright et al., 2001, showed that the atmosphere carries high concentrations of nitrate, ammonium and phosphate [25]. It is a remarkable fact that water, as rain or snow, can be characterized as a fertilizer supplying essential nutrients to aeroterrestrial algae [23]. Aeroterrestrial algal have developed, *inter alia*, thick cell walls, various compounds such as multi-functional sugar alcohols, and extracellular mucoid layers to ensure water availability [23]. In addition, the biofilm can undergo large changes in volume during dry and wet periods, or during freezing and thawing [21]. Extracellular polymeric substances (EPS), which form a coating around *Trentepohlia*, aid in the attachment of the alga to surfaces [26]. EPS also substantially increases the water holding capacity of the algal biofilm and significantly reduces water loss in algal cells exposed to periodic drying [27], [28].

Several morphological characteristics have been clearly characterized by Hoffmann [22] and Rindi et al. [10]. Liu et al. collected *Trentepohlia* in the Yajiageng valley, Mt. Gongga, and in 2012 named this alga as a new variety: *Trentepohlia jolithus* var. *yajiagengensis* var. nov. [29]. This strain has a remarkably high content of carotenoids, which helps the algal cells resist strong ultraviolet radiation at high altitudes. This variety is also rich in oils, which gives it high resistance to cold dry winters [29], [30]. The phylogenetic affinities of *Trentepohlia* and the other members of the Trentepohliales have long been unclear [10]. Perhaps as a result of global warming, epiphytic species appear to be increasing. Aptroot et al. had shown that all lichens containing *Trentepohlia* occurring in the Noordhollands Duinreservaat have increased in abundance, and in 2007 suggested that global warming might affect *Trentepohlia* directly rather than the fungal components [31]. The ecological distribution and physiological properties of a new variety of *Trentepohlia aurea*, sampled from the west plateau klint of the Sichuan Basin, China, has now been investigated in our laboratory.

High-throughput RNA-sequencing (RNA-Seq) is a recently developed technology that provides new strategies for analyzing the functional complexity of a transcriptome [32], [33]. The application of such high-throughput approaches could greatly accelerate the research progress on a new species by providing an improved understanding of essential components in a particular cellular process, the regulation of networks involved in biological processes, metabolic pathways, and responses to stimuli and so on. There is a growing number of oleaginous microalgae for which *de novo* transcriptomes have been assembled and annotated [34]–[39]. However, to date, only a few studies have been carried out in other kinds of microalgae [40]–[42]. The limited amount of molecular data available for most of the Ulvophyceae is a significant hurdle to the completion of genetic analyses and biological studies. In the present study, using Illumina sequencing and bioinformatic analysis, we have examined the *T. jolithus* transcriptome to gain more insights into its metabolic pathways. The principal objective of this study was to annotate the functional genes and identify potential metabolic pathways, such as photosynthetic carbon fixation, and fatty acid and carotenoid biosynthesis. The results provide new insights into the regulation of photosynthesis and suggest potential strategies for optimizing conditions favoring growth and carotenogenesis.

## Materials and Methods

### Ethics Statement

No specific permits were required for the described field studies, which took place in locations with public right-of-way. The field studies did not involve endangered or protected species.

### Specimen collection and RNA extraction

**Figure 1:**
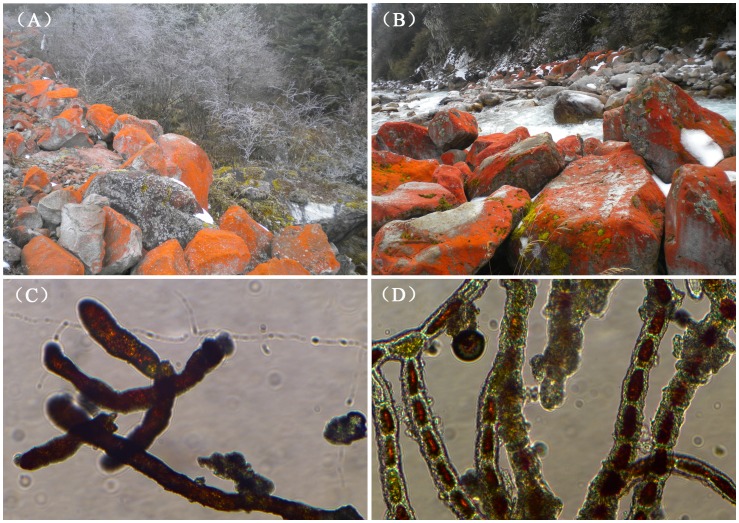
Rocks covered with red *T. jolithus*. (A) Red-Stone-Valley in winter. (B) Reddish stones along rivers. (C) Microscopic structure of dried *T. jolithus*. (D) Microscopic structure of rehydrated *T. jolithus* after a few drops of water was added to the dried material.

Specimens of *Trentepohlia jolithus* were randomly collected from reddish stones in Yanzigou, the east slope of Mt. Gongga, Luding, Sichuan province (China; 29°38′N, 102°7′E) on Dec 1, 2012 (Figure 1 A). According to the weather station at 3000 m, temperature, air humidity and light intensity exhibit diurnal and seasonal fluctuations. The mean temperature we measured during daytime varied from 12°C to–4°C at 3100 meters above sea level. This area has a very mild climate with an average relative humidity above 90%, which is ideal for *Trentepohlia* growth [43]. The light intensity we measured during daytime ranged from 30 to 2500 µmol photons m^−2^ s^−1^ depending on the degree of cloud cover. When we were sampling at noon, the temperature, humidity and light intensity were 7°C, 51% and 2200 µmol photons m^−2^ s^−1^, respectively. These epilithic algae colonize the exposed rock surface and extend for a few tens of kilometers along the valley. The filamentous green algae form large, bright red to deep red biofilms on the rocks (Figure 1 A, B). Samples of the wild algae were carefully scraped from the stones with sharp knives under the existing environmental conditions. Several pieces of *T. jolithus* were selected based on their bright color and apparent cleanness. To maximize information on variability, *T. jolithus* samples from different heights were chosen for the experiment and sealed in plastic collection bags. The materials were kept in a car refrigerator and sent to our laboratory in Qingdao by air.

In the laboratory, *T. jolithus* cells were harvested after washing and filtration to remove visible contaminants. First, the samples were rehydrated, a process that takes just a few seconds (Figs 1 B and 1 C). Mortars and a 70-µm sieve cloth were used to break up the biofilm gently during the washing process, using sterilized water. Then, a piece of algal material obtained by filtration through the sieve cloth at room temperature, was resuspended in sterile deionized water, and transferred into a new mortar. After washing several times with the sterilized water, any remaining lichen material and other contaminants were removed and clean unialgal samples were selected. Even so, there is a possibility that we sampled a community and that this transcriptome sample was a metatranscriptome greatly enriched for *T. jolithus*. The identity of the organism was further confirmed from these samples by careful examination of the morphology under a microscope and by 18S rDNA sequencing. The bulk of the clean selected samples was dried with hygroscopic filter paper and flash frozen immediately in liquid nitrogen for subsequent RNA extraction.

### Preparation of total RNA

RNA from an 80 mg subsample was extracted and purified using an Plant RNA Kit following the manufacturer’s instructions. The RNase-free DNase I digestion protocol was performed to remove residual genomic DNA. Quantity and purity of the extracted RNA was determined by a Nanodrop ND-100 spectrophotometer (LabTech, Holliston, MA, USA). RNA integrity was confirmed via an Agilent 2100 bioanalyzer (Agilent; Palo Alto, CA, USA), which gave an RNA integrity number (RIN) of 7.9.

### RNA-Seq Library Preparation and Sequencing

Four µg of the total RNA sample were used to isolate poly-A containing mRNA molecules, using poly-T oligo-attached magnetic beads (Illumina). Then, the poly-A RNA was purified and fragmented into smaller pieces (200–700 nt) using divalent cations at 94°C for exactly 5 minutes. This protocol uses two rounds of enrichment for poly-A mRNA followed by thermal mRNA fragmentation. Aliquots of purified mRNA were used for construction of the cDNA libraries using the mRNA-Seq Kit supplied by Illumina. During the cDNA synthesis process, the cleaved RNA fragments were primed with random hexamers and then reverse-transcribed into first strand cDNA using reverse transcriptase and random primers. The cDNA was next converted into double stranded DNA using the reagents supplied in the Illumina TruSeq RNA sample preparation kit, according to the manufacturer’s protocol, and the resulting dsDNA was used for library preparation. After adenylating the 3′ ends and ligating adapters to the ends of the dscDNA, we selectively enriched for those DNA fragments that had adapter molecules on both ends, and amplified the amount of DNA in the library using PCR. During the QC (Quality Control) steps, an Agilent 2100 Bioanalyzer and an ABI StepOnePlus Real-Time PCR System were used for quality assessment and quantification of the sample library. Finally, the library was sequenced using an Illumina HiSeq 2000, at Huada Genomics Institute Co. Ltd, China. After the sequencing was completed, the image data output was transformed by base calling into sequence data; this output was called “raw data” or “raw reads” and stored in fastq format. Raw sequence data has been submitted to the NCBI Sequence Read Archive (SRA) with the accession number SRP033549.

### Raw Data Analysis and *De Novo* Transcriptome Assembly

Before assembly, the raw reads were filtered to obtain high-quality clean reads. Dirty raw reads with adaptor sequences, with unidentified nucleotides in excess of 5%, as well as low quality reads having more than 20% low quality base identification (base quality <10) were discarded. At this point, 90 bp paired-end reads were extracted from the raw data. *De novo* assembly of the clean reads was performed using Trinity software (release-20121005) as described for *de novo* transcriptome assembly without reference genome [44]. The sequences resulting from the trinity assembly were called unigenes.

### Unigene annotation and classification

For unigene functional annotation, BLAST alignments (E-value <10^−5^) were carried out using unigene sequences to query databases. The queried databases included the NCBI non-redundant protein database (NR), Kyoto Encyclopedia of Genes and Genomes (KEGG), Swiss-Prot, and Clusters of Orthologous Groups (COG). When a unigene did not align with anything in the above databases, software named ESTScan [45] was introduced to decide its sequence direction. Gene ontology (GO) assignments were applied using the Blast2go program for functional annotation [46]. WEGO software [47] was used to do GO functional classification. Pathway assignments were determined following the KEGG pathway database using BlastX with an E-value threshold of 1.0E-5. Simple sequence repeat (SSR) detection was done with MIcroSAtellite (MISA) software using unigenes as reference. For SNP analysis, SNPs were detected using SoapSNP (http://soap.genomics.org.cn/soapsnp.html) essentially as described by Li et al. [48].

### Pigment measurements and microscopic observation

Chlorophylls and carotenoids were extracted by 80% acetone and analyzed with a UV-120 system (Shimadzu, Japan) as described by Şükran et al. [49]. The microscopic structure of the friable dried material was photographed using an inverted biological microscope (BM-37XB). When a few drops of water were added, the materials exhibited a strong water absorbency, which was observed by microscope, and photographed after a few seconds, in the same way.

## Results and Discussion

### Illumina sequencing and read assembly

**Figure 2:**
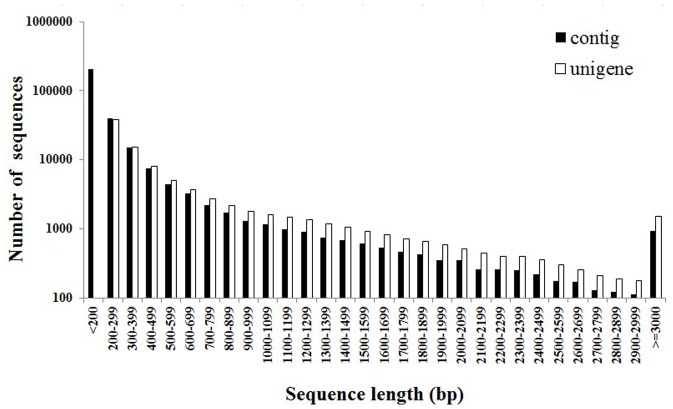
Length distribution of assembled contigs and unigenes.

In total, 55,007,830 Illumina PE raw reads were obtained. After removing reads with adapters and unknown or low quality bases, approximately 52.39 million clean reads with an average length of 90 bp were obtained (97.95% Q20 bases and 39.88% GC content). The complete assembly yielded 292,122 contigs with a mean length of 250 bp. The raw assembly contigs were clustered into a unigene dataset, which resulted in 92,414 unigenes that ranged from 200 bp to 9198 bp with a mean length of 619 bp and an N50 length of 986 bp (Table 1). The length distribution of the contigs and unigenes are illustrated in Figure 2.

**Table 1:** Summary of sequence analysis.

Description	Number (n)	Bases (bp)
**Sequencing**		
Total raw reads	55,007,830	
Total clean reads	52,390,656 4,715,159,040	
Q20 percentage (%)	97.95	
N percentage (%)	0.02	
GC content (%)	39.88	
**Contigs**		
Total contigs	292,122	73,002,694
Min length (bp)	200	
Max length (bp)	9198	
Average length (bp)	250	
N50 (bp)	287	
**Unigenes**		
Total unigenes	92,414	57,175,212
Min length (bp)	200	
Max length (bp)	8785	
Average length (bp)	619	
N50 (bp)	986	

### Functional annotation and classification

**Figure 3:**
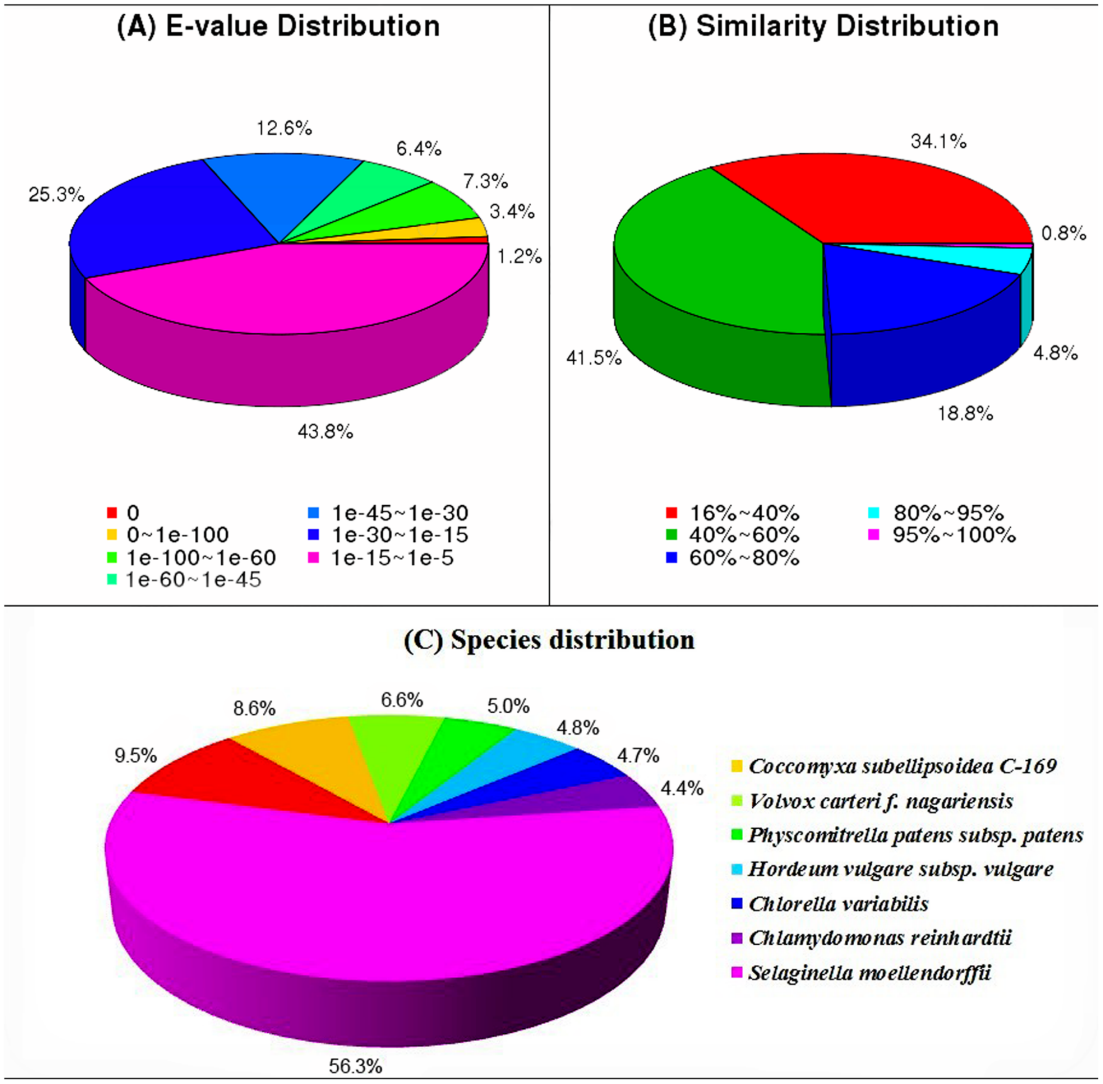
Characteristics of similarity search of unigenes against NR database. (A) E-value distribution of BLAST hits for each unigene with a cutoff E-value of 1.0E-5. (B) Similarity distribution of the top BLAST hit for each unigene. (C) Species distribution of the top BLAST hit for each unigene in the NR database.

For assignments of predicted genes, 41,328 assembled unigenes were obtained after annotations with the NR, NT, Swissprot, KEGG, COG and GO databases, using the BLAST algorithm, specifying E values of less than 10^−5^. The results indicated that 37,869 (91.63%) and 9,674 (23.41%) non-redundant unigenes showed identity with sequences in NCBI NR and NT databases, respectively. Of the 23,274 annotated unigenes, 56.32% had similarity to proteins in the Swiss-Prot database (Table 2). E-value and similarity distributions of the top hits in the NR database analysis revealed that 18.36% (6,950) and 5.54% (2,100) showed significant homology (E-value <1E-45) or high similarity (greater than 80%), respectively (Figure 3 A, B). About 43.66% of annotated unigenes could be assigned with a best score to the sequences from the top seven species, i.e., *Coccomyxa subellipsoidea* C-169 (9.48%), *Volvox carteri f. nagariensis* (8.56%), *Physcomitrella patens subsp. patens* (6.59%), *Hordeum vulgare subsp. vulgare* (5.03%), *Chlorella variabilis* (4.83%), *Chlamydomonas reinhardtii* (4.73%) and *Selaginella moellendorffii* (4.44%) (Figure 3 C).

**Table 2:** BLAST analysis of non-redundant unigenes against public databases.

Database	Number of annotated unigenes	Percentage of annotated unigenes
**NR**	37,869	91.63%
**NT**	9,674	23.41%
**Swiss-Prot**	23,274	56.32%
**KEGG**	26,217	63.44%
**GO**	22,018	53.28%
**COG**	22,921	55.46%

### Functional annotation and pathway assignment

**Figure 4:**
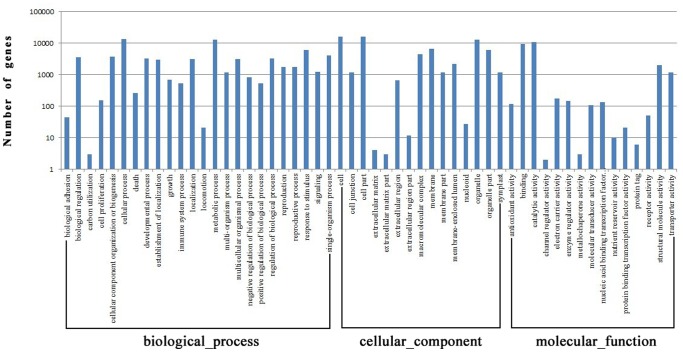
Gene Ontology classification of assembled unigenes. The results are summarized in three main categories: biological process, cellular component and molecular function. In total, 22,018 unigenes with BLAST matches to known proteins were assigned to this gene ontology.

Based on NR annotation, 53.28% of unigenes (22,018) were assigned to gene ontology classes with 54 subcategories and 161,451 functional terms (Figure 4, Table S1). Biological process (68,837; 42.64%) and cellular component (68,184; 42.23%) comprised the majority of the functional terms. Additionally, 24,430 unigenes (15.13%) were classified into molecular function categories. Within the biological process category, cellular process (19.74%) was the most dominant group, followed by metabolic process (18.71%) and response to stimulus (8.96%). In the category of cellular component, cell and cell part were the most highly represented groups with the same percentage of 23.33%. Regarding molecular function, most of the corresponding genes were assigned to catalytic activity (44.03%) and binding (39.55%).

**Figure 5:**
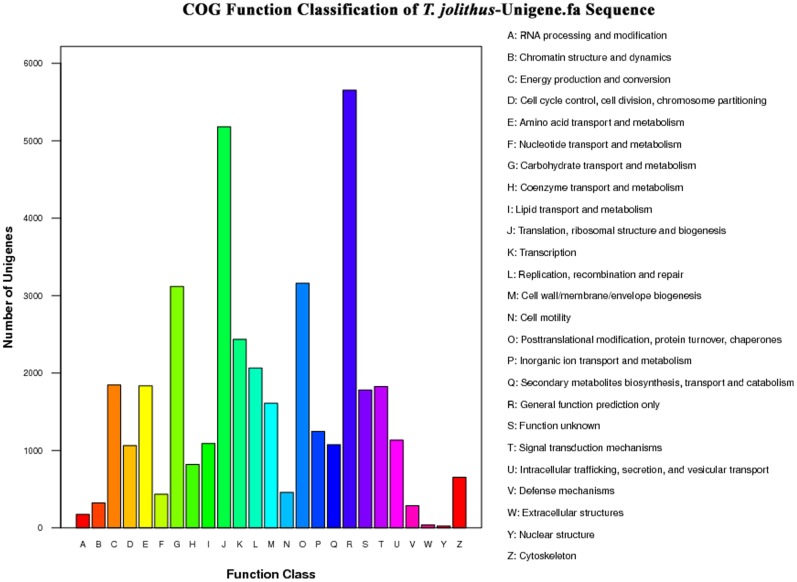
Clusters of orthologous groups (COG) functional classification. In total, 22,921 of the 41,328 sequences with an NR hit were grouped into 25 COG classifications.

Clusters of Orthologous Groups of proteins (COGs) were delineated by comparing protein sequences encoded in complete genomes, representing major phylogenetic lineages. The COG database was used to define the orthologous functions of unigenes. In total, 22,921 (55.46%) of the non-redundant unigenes (Table 2) were subdivided into 25 COG classifications. The top categories among the COG terms were ‘General function prediction only’ (5,652; 14.38%) and ‘Translation, ribosomal structure and biogenesis’ (5,180; 13.18%). Whereas ‘Extracellular structures’ (37; 0.09%) and ‘Nuclear structure’ (22; 0.06%) were poorly characterized (Figure 5, Table S2).

The Kyoto Encyclopedia of Genes and Genomes (KEGG) database [50] was used to identify the biological pathways in *T. jolithus*. A total of 26,217 (63.44%) of the assembled unigenes were mapped to 128 KEGG pathways (Table S3). Highly represented pathways included metabolic pathways (6357 members), biosynthesis of secondary metabolites (3058 members), ribosome (2571 members) and spliceosome (1839 members). Additionally, terpenoid backbone biosynthesis (82 members), flavonoid biosynthesis (79 members), and fatty acid biosynthesis (106 members) were identified as potential pathways that need follow-up studies for confirmation.

### EST-SSR Discovery, Distribution and Frequencies

**Figure 6:**
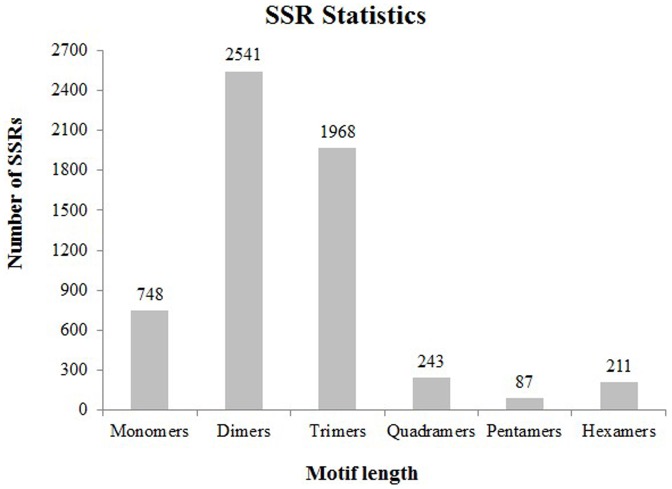
Distribution of identified SSRs using MISA software.

Simple Sequence Repeats (SSRs), also known as microsatellites, have been developed as SSR markers for genomic mapping, DNA fingerprinting, and marker-assisted selection in many species [51], [52]. They are tandemly repeated sequences comprised of 1–6 base pairs of DNA, with a conserved flanking sequence [53]. In our study, all of the 92,414 unigenes generated were used to mine potential microsatellites using MISA software. 6,187 SSR primer pairs were designed from these loci and a total of 5,798 SSRs were identified in 5,206 unigenes. Of all the SSR containing unigenes, 527 sequences contained more than one SSR and 256 SSRs were present in compound form. Frequencies for each array type according to repeat motifs are illustrated in Figure 6, the most abundant being dimers (43.83%), followed by trimers (33.94%), monomers (12.90%), quadramers (4.19%), hexamers (3.64%) and pentamers (1.50%). SSRs with six tandem repeats (22.47%) were the most common, followed by five tandem repeats (21.70%), seven tandem repeats (15.04%), and nine tandem repeats (7.49%). A summary of the number of repeat units is available in Table S4. These SSR results provide useful new molecular markers for any future genetic linkage analyses in *T. jolithus*.

### SNP detection

Single-nucleotide polymorphisms (SNP) were identified as heterozygous sites on the transcripts, using SOAPsnp software [48]. A total of 131,478 putative SNPs were predicted with default parameter values as shown in Table 3 and Table S5. Within the detected SNPs, transitions (66.42%) were much more common than transversions (33.58%). The number of A-G transitions (43,348) was similar to that for C-T (43,979). Among the transversions, A-T (47.91%) dominated, followed by A-C (20.03%), G-T (19.36%) and C-G (12.69%). Considering their abundance and variety, it would be possible to use these SNP-based markers to generate dense genetic maps and, in the future, to perform marker assisted selection (MAS) as described by Barbazuk et al. [54]. This SNP database also offers rich information on the diversity within the species and will be used to study remaining uncertainties about *T. jolithus* strain distribution. It was worth cautioning at this point that there is a possibility that multiple organisms from the original community contributed to the sequences and thus the genomic diversity reported here will need further confirmation that it is truly derived from the *T. jolithus* genome.

**Table 3:** Summary of putative SNPs found in *T. jolithus* unigenes.

SNP Type	Count
**Transition**	87,327 (66.42%)
A-G	43,348
C-T	43,979
**Transversion**	44,151 (33.58%)
A-C	8,845
A-T	21,153
C-G	5,604
G-T	8,549
**Total**	131,478

### Pigment content and microscopic structures of *T. jolithus*

The chlorophyll level in the tested *T. jolithus* varied from 0.63%–0.70% with an average of 0.67% of dry weight (DW). Carotenoids accounted for 2.2% of DW, which was 3.30 times the level of chlorophyll (Table 4). These high levels of carotenoids would effectively protect the cells from photodamage in the high altitude valleys, where the alga is found. An investigation of the “Red-Stone-Valley” habitat showed that the alga formed an extensive red covering on exposed rocks in winter (Figure 1 A). It was typically distributed on rocks that are located away from the river. The alga is not found on rocks located in or near the river and are periodically submerged. This observation is consistent with the fact that aeroterrestrial phototrophic microorganisms typically form conspicuous biofilms at the interface between any type of solid substratum and the atmosphere (Figure 1 B). Under the microscope, erect filaments were clearly observed in the dried materials (Figure 1 C) of the alga. After absorbing water rapidly, shiny red, individual cells were evident in the filaments, in contrast to the more amorphous appearance of filaments in the dried materials (Figure 1 D).

**Table 4:** Determination of carotenoid and chlorophyll contents in *T. jolithus*.

Type	Average (% of DW)	Range (% of DW)	Ratio (Car/Chl)
**Carotenoid**	2.20	1.99–2.41	3.30
**Chlorophyll**	0.67	0.63–0.70	

### 18S small subunit ribosomal DNA gene

In addition to observing the microscopic structures of dried and rehydrated *T. jolithus* (Figure 1 C and D), we sequenced the 18S rDNA to avoid taxonomic confusion. PCR amplification and sequencing of *Trentepohlia jolithus* 18S rDNA were performed using primers designed for both algae and plants. The sequence was deposited with GenBank under the Accession no. KM112092. BLAST analysis indicated that the sequence was homologous to previously identified *Trentepohlia*. According to the results, we aligned the 18S rDNA sequence with previous reported *Trentepohlia jolithus* var. *yajiagengensis* var. nov (JN542473), which was also collected from Sichuan province in China [29]. We found just two differences among 1612 bases and showed 99.88% sequence similarity (Figure S1).

### Analysis of genes and pathways

Although there are over 40,000 species of microalgae identified to date, the genomes of fewer than a dozen of these have been sequenced (http://genome.jgi-psf.org). Overall, biological pathways in microalgae are far from fully documented. Because there is extensive genomic sequence data for model organisms such as *Chlamydomonas reinhardtii*, these organisms are best for carrying out research at the transcriptomics and proteomics level to study responses to variables such as physiological stress at the molecular level [55]–[57]. For less known organisms, transcriptome sequencing and comparison with known organisms can be an efficient approach for obtaining a great deal of functional genomics information, thereby gaining information about additional organisms and reducing the reliance on model organisms [58]. In this connection, a few molecular and biochemical studies have been carried out in the Trentepohliaceae [8], [11], but there is still a very limited understanding of the metabolic pathways, and of mechanisms controlling growth. In the present study, we obtained a large number of cDNA fragments that were highly enriched in genes for carbon fixation and metabolic pathways representative of the order Trentepohliales. Among these, we focused on key genes involved in carbon fixation, and in fatty acid and carotenoid biosynthesis. The principal components of these metabolic pathways are described below.

### Carbon fixation

From many studies on primary photosynthetic carbon metabolism, it was believed that the operation of the Calvin–Benson cycle (C3 pathway) was predominant in algae [59]. The initial carbon fixation catalyzed by Rubisco produces two three-carbon molecules of 3-phosphoglyceric acid (3-PGA) through the carboxylation of the five-carbon ribulose-1,5-biphosphate (RuBP) [60]. In addition, microalgae increase the level of CO_2_ by accumulating HCO_3_^−^ to overcome Rubisco’s surprisingly poor affinity for CO_2_ and prevent the diffusion of CO_2_ out of the cell, while allowing the entry of other nutrients [61]. However, recent metabolic labeling and genome sequencing data suggest that they may also perform C4 photosynthesis. In our study, both C3 and C4 photosynthesis genes were found by transcriptome sequencing. Together these results suggest that C4 photosynthesis might be more wide-spread than previously thought.

**Figure 7:**
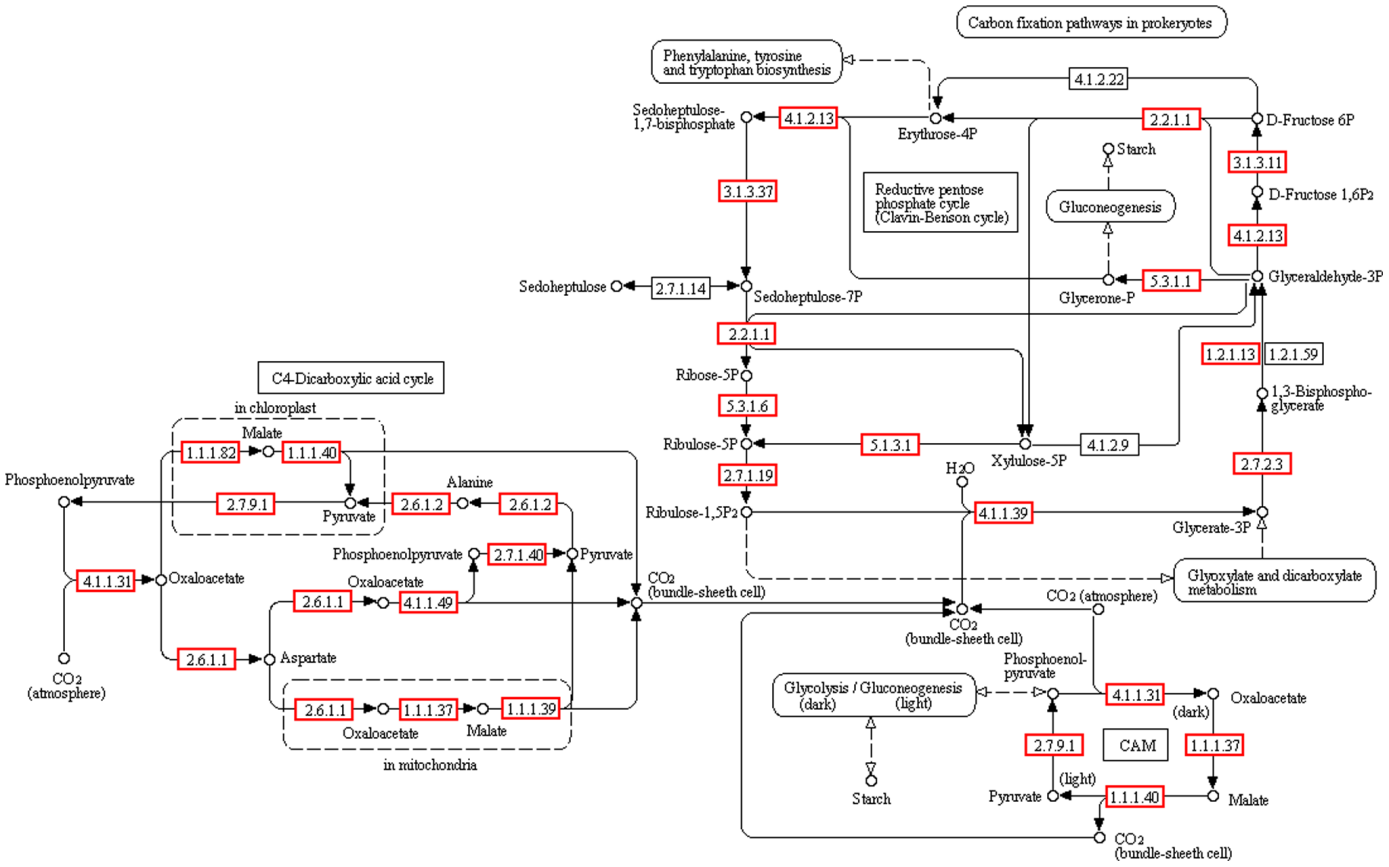
Putative pathway of carbon fixation in *T. jolithus*, generated by KEGG. The numbers within the small boxes are enzyme codes. The boxes with a red border are enzymes identified in this study. The boxes with a black border are enzymes not identified in this study.

In our study, using BLAST against the KEGG database, most of the genes for the key enzymes related to the C3 (139 unigenes) and C4 (157 unigenes) pathways of carbon fixation were actively transcribed (Figure 7, Table S6). Eleven transcripts (including 9 separate unigenes) of ribulose 1,5-bisphosphate carboxylase/oxygenase (Rubisco), which is the central carboxylation enzyme for CO_2_ fixation during photosynthesis, were obtained from *T. jolithus.* We also identified most of the genes involved in the Calvin-Benson cycle, such as fructose-bisphosphate aldolase (EC 4.1.2.13), phosphoglycerate kinase (EC 2.7.2.3), triose-phosphate isomerase (EC 5.3.1.1) and so on. These results provide unequivocal molecular evidence for the existence of a C3-pathway in *T. jolithus.*

It was interesting that enzymes required for C4 photosynthesis were also identified (Figure 7, Table S6). Among these genes, pyruvate orthophosphate dikinase (PPDK) is an important C4 enzyme that is required for the regeneration of phosphoenolpyruvate (PEP) in mesophyll cells and is regulated at the transcriptional level [62]. Phosphoenolpyruvate carboxykinase (PEPCK) is another key enzyme and used to catalyze the release of CO_2_ from oxaloacetate (OAA) to produce PEP in the bundle sheath cell cytosol [60]. In addition to this, PPDK and PEPCK are also present in C3/CAM species [63], [64]. Parsley et al. had proposed that in cotyledons PPDK may be important in supplying PEP for gluconeogenesis, and in ageing leaves it allows remobilization of nitrogen to supply reproductive tissue among C3 plants [63]. Its activity is light/dark modulated by reversible phosphorylation in both C3 and C4 plants [65]. PEPCK also supplies CO_2_ for fixation by Rubisco during the light period in some CAM species [66]. In the present study, by transcriptome sequencing and gene annotation, 10 PPDK and 16 PEPCK transcripts were identified. The function of these two genes warrants further investigation. Transcriptome information about C4-related enzyme variations among various algae is considerable. In the Bacillariophyta, *Phaeodactylum tricornutum* [67] and *Thalassiosira pseudonana* [68] have been shown to have developed a C4-like photosynthesis pathway in addition to the carbon-concentrating mechanism (CCM). In the Rhodophyta, almost all of the key enzymes in the C4 carbon-fixation pathway have been detected; however, PEPC in *Pyropia* (*Porphyra) haitanensis* sporophytes and pyruvate phosphate dikinase (EC 2.7.9.1) in *Pyropia* (*Porphyra) yezoensis* were not detected among the ESTs or transcriptome respectively. In the Chlorophyta, *Ulva linza* and *Ulva prolifera* have been shown to contain the C4 pathway [69]. *Myrmecia incisa* Reisigl H4301, a coccoid green microalgal species belonging to the Trebouxiophyceae [70], *Ostreococcus tauri,* the smallest free-living eukaryote [71] and *Micromonas* sp., a marine picoeukaryote [72] also possibly possesses a C4-like photosynthesis pathway. Despite all this, a short-term metabolic ^14^C labeling experiment carried out on *Thalassiosira weissflogii* and *Thalassiosira pseudonana* by Roberts et al. suggested that intermediate compounds during the carbon assimilation of photosynthetic pathways were diverse [73]. Hence, our transcriptome data and literature on the related organisms [67]–[72] suggest that *T. jolithus* might possess a complex carbon acquisition and fixation system without loss of CCM. This effective mechanism could help *T. jolithus* cope with high light intensities, low temperatures and arid conditions [60], [74]. It also emphasizes the requirement for metabolic and functional genetic analyses before accepting the presence of C4-metabolic enzymes as evidence for C4 photosynthesis.

### Fatty acid biosynthesis

Fatty acids, which are the building blocks for the formation of various types of lipids, are a potential biofuel feedstock [75]. The basic pathway for fatty acid biosynthesis in microalgae is found primarily in the chloroplast, and is generally believed to be analogous to the pathway found in higher plants [76]. Based on the functional annotation of the transcriptome, we have successfully identified the genes encoding key enzymes involved in the biosynthesis and catabolism of fatty acids in *T. jolithus* (Table S8). Fatty acid biosynthesis of this species starts with the conversion of acetyl CoA to malonyl CoA, catalyzed by acetyl CoA carboxylase (ACCase, EC: 6.4.1.2), which is consistent with *Dunaliella tertiolecta* and *Eustigmatos* cf. *polyphem*. Then, malonyl-CoA, the central carbon donor for fatty acid synthesis, is transferred to malonyl-acyl carrier protein (ACP) catalyzed by long-chain acyl-CoA synthetase (FabD, EC: 6.2.1.3). The number of transcripts involved in all elongation reactions of this pathway were 4, 66, 1 and 2 for 3-Ketoacyl ACP synthase II (FabF, EC 2.3.1.179), 3-oxoacyl-ACP reductase (FabG, EC 1.1.1.100), 3-Hydroxy acyl-CoA dehydratase (FabZ, EC 4.2.1.59) and enoyl-ACP reductase I (FabI, EC 1.3.1.9 1.3.1.10), respectively. Hexadecenoic and Octadecanoic acid were finally synthesized by fatty acyl-ACP thioesterase A (FATA, EC 3.1.2.14 3.1.2.−) and oleoyl-ACP hydrolase (OAT, EC 3.1.2.14) catalysis. These C16 and C18 trienoic fatty acids could be used as the precursors for the synthesis of cellular membranes, long-chain polyunsaturated fatty acids (LC-PUFAs) and storage neutral lipids (mainly TAGs) [36]. The PUFAs may enhance the fluidity of the phospholipid membrane, which makes it possible for *T. jolithus* to withstand chilling or cold stress in the alpine environment [77].

### Carotenoid biosynthesis

Carotenoids play an important role as light-harvesting pigments and protect the photosynthetic apparatus from photooxidative damage under excess light conditions [78]. In 2011, Takaichi summarized 28 different structures of carotenoids from the algal species he studied [79]. Previously, carotenogenesis pathways and their enzymes in oxygenic phototrophs had been investigated in cyanobacteria [80] and land plants [81]. Microalgae had common pathways with land plants and also additional microalgal-specific pathways and carotenoids [70]. Many carotenogenesis enzymes and genes such as CrtB, CrtP, CrtL-b, CrtR-b, ZEP, VDE, and CrtW were reported in the Chlorophyceae, including *Chlorella*, *Chlamydomonas*, *Dunaliella* and *Haematococcus* [79]. Nevertheless, only a little was learned about the carotenogenesis pathways of algae except for some proposed chemical structures [79]. The kinds and amounts of various carotenoids in *Trentepohlia* have been studied. High light intensity has been shown to be an important factor for increasing carotenoid levels in *Trentepohlia aurea* and *Trentepohlia odorata* by Abe et al. [82] and Tan et al. [83], respectively. Mukherjee et al. also found that carotenoid content increased several fold seasonally, possibly because the reduced local temperatures (∼25°C) and cloudless clear skies in winter provide ideal conditions favoring growth and carotenogenesis in two tropical species, *T. aurea* and *T. cucullata* [84]. In this study, the high levels of carotenoids convincingly demonstrate the existence of a carotenoid biosynthesis pathway in *T. jolithus*. Sequence information and pathway analysis will facilitate molecular functional studies and pave the path for a better understanding of carotenogenesis pathways in this aerial microalga.

**Figure 8:**
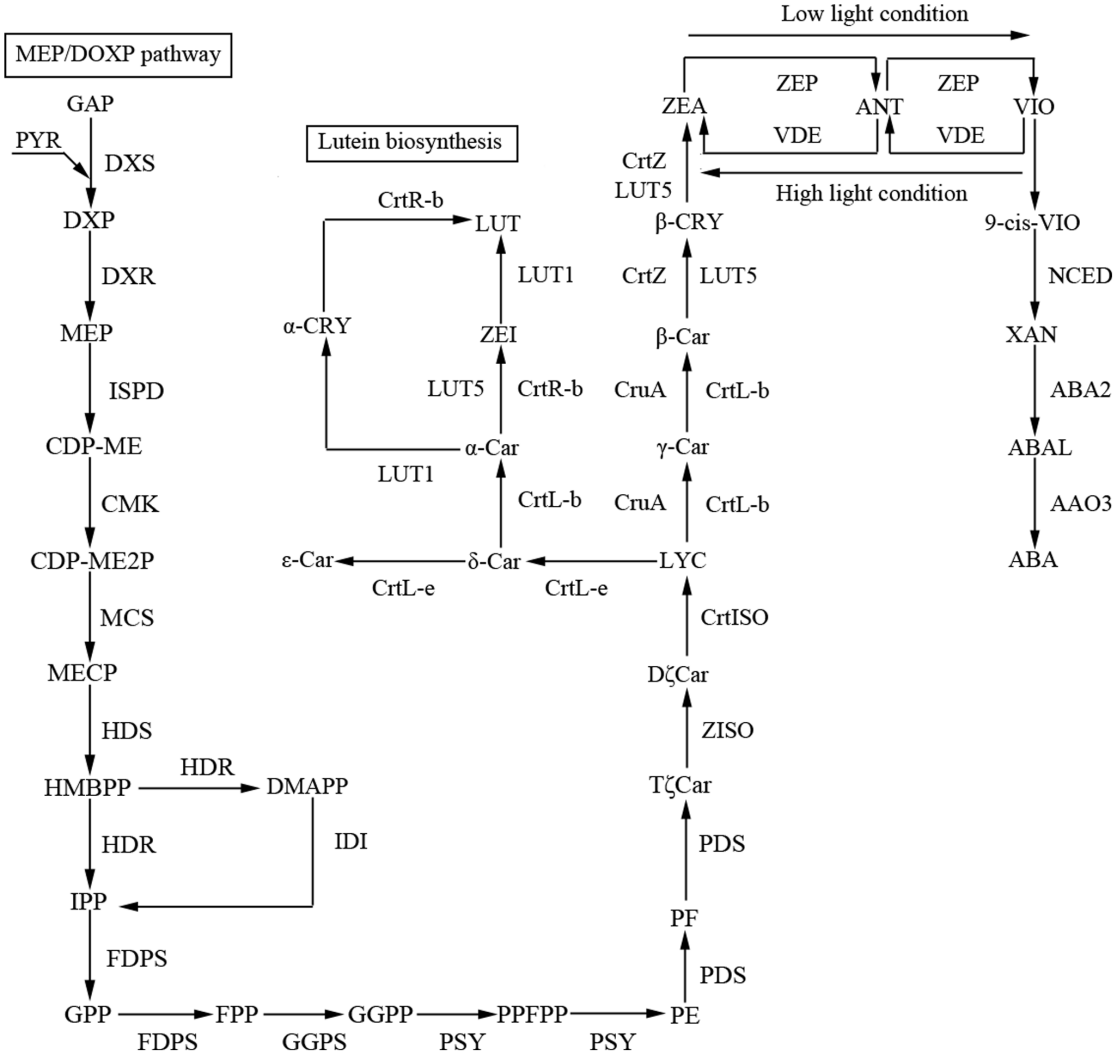
Carotenoid biosynthesis model for *T. jolithus* based on annotations in the transcriptome library. Abbreviations of chemical compounds are as follows: GAP, D-glyceraldehyde-3-phosphate; PYR, pyruvate; DXP, 1-Deoxy-D-xylulose 5-phosphate; MEP, 2-C-Methyl-D-erythritol 4-phosphate; CDP-ME, 4-(Cytidine 5′-diphospho)-2-C-methyl-D-erythritol; CDP-ME2P, 2-Phospho-4-(Cytidine 5′-diphospho)-2-C-methyl-D-erythritol; MECP, 2C-methyl-D-erythritol-2,4-cyclodiphosphate; HMBPP, 1-Hydroxy-2-methyl-2-butenyl 4-diphosphate; DMAPP, Dimethylallyl-PP; IPP, Isopentenyl-PP; GPP, Geranyl-PP; FPP, (E,E)-Farnesyl-PP; GGPP, Geranylgeranyl-PP; PPFPP, prephytoene diphosphate; PE, phytoene; PF, 15,9′-dicis-Phytofluene; TζCar, 9,15,9′-tricis-ζ-Carotene; DζCar, 9,9′-Di-cis-ζ-Carotene; LYC, Lycopene; β-CRY, β-Cryptoxanthin; ZEA, Zeaxanthin; ANT, Antheraxanthin; VIO, Violaxanthin; 9-cis-VIO, 9-cis-Violaxanthin; XAN, Xanthoxin; ABAL, Abscisic aldehyde; ABA, Abscisate; α-CRY, α-Cryptoxanthin; ZEI, Zeinoxanthin; LUT, Lutein; α-Car, α-carotene; β-Car, β-carotene; γ-Car, γ-carotene; δ-Car, δ-carotene; ε-Car, ε-carotene. Abbreviations of enzymes are as follows: DXS, 1-deoxy-D-xylulose-5-phosphate synthase; DXR, 1-deoxy-D-xylulose-5-phosphate reductoisomerase; ISPD, 2-C-methyl-D-erythritol 4-phosphate cytidylyltransferase; CMK, 4-diphosphocytidyl-2-C-methyl-D-erythritol kinase; MCS, 2-C-methyl-D-erythritol 2,4-cyclodiphosphate synthase; HDS, (E)-4-hydroxy-3-methylbut-2-enyl-diphosphate synthase; HDR, 4-hydroxy-3-methylbut-2-enyl diphosphate reductase; IDI, isopentenyl-diphosphate delta-isomerase; FDPS, farnesyl diphosphate synthase; GGPS, geranylgeranyl diphosphate synthase, type III; PSY, phytoene synthase; PDS, 15-cis-phytoene desaturase; ZISO, zeta-carotene isomerase; CrtISO, prolycopene isomerase; CrtR-b, lycopene beta-cyclase; CruA, lycopene cyclase CruA; CrtL-b, lycopene beta-cyclase; CrtL-e, lycopene epsilon-cyclase; LUT5, cytochrome P450, family 97, subfamily A (beta-ring hydroxylase); CrtZ, beta-carotene 3-hydroxylase; LUT1, carotene epsilon-monooxygenase; ZEP, zeaxanthin epoxidase; VDE, violaxanthin de-epoxidase; NCED, 9-cis-epoxycarotenoid dioxygenase; ABA2, xanthoxin dehydrogenase; AAO3, abscisic-aldehyde oxidase.

Based on the functional annotation of the transcriptome, we have successfully identified 99 unigenes encoding key enzymes involved in carotenogenesis in *T. jolithus* (Figure 8, Table S7). The mevalonate (MVA) pathway beginning with Acetyl-CoA in plant cytoplasm, and the MEP/DOXP pathway, beginning with pyruvate in the plastids of algae, are two known independent pathways for IPP synthesis [79]. There were no identified unique sequences encoding mevalonate kinase (MVK, EC 2.7.1.36), which is required for the former pathway. MVK, phosphomevalonate kinase (PMVK, EC 2.7.4.2) and mevalonate pyrophosphate decarboxylase (MPD, EC 4.1.1.33) were also absent in *Myrmecia incisa* Reisigl H4301, a coccoid green microalgal species in the Trebouxiophyceae [70]. The result of annotated unigenes was consistent with the lack of an MVA pathway in the Chlorophyceae [85]. In contrast, all the genes encoding enzymes involved in the MEP/DOXP pathway have been identified. After one IPP was added to farnesyl pyrophosphate by two transcripts of geranylgeranyl pyrophosphate synthase, type III (GGPS, EC 2.5.1.29), geranylgeranyl pyrophosphate (GGPP) was produced and acted as the substrate for phytoene synthase (PSY, EC 2.5.1.32), encoded by another unigene, which is the same as that in *E.* cf. *polyphem* [36]. Following the formation of phytoene (C40), four steps were needed in the conversion from phytoene to lycopene by the sequential catalysis of phytoene desaturase (PDS, EC 1.3.5.5), ζ-carotene isomerase (ZISO, EC 5.2.1.12), ζ-carotene desaturase (ZDS, EC 1.3.5.6) and carotenoid isomerase (CrtISO, EC 5.2.1.13) [70]. In the current study on *T. jolithus*, the number of transcripts in the transcriptome library coding for the enzymes involved in these four steps was one for PSY, two for PDS, one for ZISO and four for CrtISO. Unlike *E.* cf. *polyphem* and *M. incisa*, ZISO involved a single isomerization instead of two desaturation steps worked by ZDS. Therefore, we could properly deduce that ZISO is a unique gene present in *Trentepohlia*. Subsequently, lycopene is an important intermediate in the biosynthesis and could be cyclized into either β-carotene through γ-carotene, or α-carotene through δ-carotene. In this study, one transcript coding for lycopene β-cyclase (CrtL-b, EC 5.5.1.19) and another for lycopene ε-cyclase (CrtL-e, EC 5.5.1.18) sufficiently confirmed the presence of β-carotene and α-carotene, which is similar to *M. incisa* [70] and *Prochlorococcus marinus* MED4 [86]. In addition, CruA, which belongs to a new family of functional lycopene cyclases, has been found in this study and cyclizes lycopene into β-carotene through γ-carotene, the same as CrtL-b. By comparison, *T. jolithus* is the first Chlorophyte species that has been shown to use CruA, which suggests it could be used as a chemotaxonomic marker. Next, lutein biosynthesis in *T. jolithus* starts with the conversion of α-carotene to lutein by LUT5, CrtR-b and LUT1. On the other hand, β-carotene is hydroxylated by β-carotene 3-hydroxylase (CrtZ, EC: 1.14.13.129) and LUT5 to form zeaxanthin through β-cryptoxanthin. The transformation between zeaxanthin and violaxanthin is carried out by zeaxanthin epoxidase (ZEP, EC: 1.14.13.90) and violaxanthin deepoxidase (VDE, EC: 1.10.99.3) respectively, under low and high light conditions with antheraxanthin as the intermediate. All the genes encoding enzymes including 9-cis-epoxycarotenoid dioxygenase (NCED, EC 1.13.11.51), abscisic-aldehyde oxidase (AAO3, EC 1.2.3.14) and xanthoxin dehydrogenase (ABA2, EC 1.1.1.288) involved in the ABA biosynthesis were detected, suggesting that ABA biosynthesis pathways in this alga are the same as *E.* cf. *polyphem* [36]. Carotene β-ketolase (CrtO) was not detected in our study and if it is truly missing from the genome, its lack would hinder or preclude the synthesis of astaxanthin by this species.

## Conclusions

In this study, which presents the first transcriptomic study within the Trentepohliales, 41,328 assembled unigenes was obtained. The functional annotation and classification of these unigenes has given us a better understanding of the *T. jolithus* genome. After analyzing typical KEGG pathways, we discovered similarities and differences between *T. jolithus* and other algae. Our results help us explain why this aerial microalgae can survive and spread in such a cold, high altitude habitat. These results also pave the way for more detailed investigations of the mechanisms underlying the growth and metabolism of members of the Trentepohliaceae. The adaption mechanisms of *T. jolithus* to desiccation and a cold environment need further investigation. Molecular genetic manipulation of this organism might be an effective way to enhance the properties of this microalga to make it suitable for commercial development.

## Supporting Information

Figure S1 **Alignment of 18S ribosomal DNA sequence of** ***T. jolithus*** **(KM112092) with** ***Trentepohlia jolithus*** **var.** ***yajiagengensis*** **var. nov (JN542473).** Identical bases were shaded in light grey, and different bases were shaded in black.
(TIF)

Table S1 **List of** ***T. jolithus*** **unigenes in each GO category.**
(XLS)

Table S2 **List of** ***T. jolithus*** **unigenes in each COG category.**
(XLS)

Table S3 **128 KEGG pathways with pathway ID and KO information.**
(XLS)

Table S4 **Summary of the number of repeat units identified from the** ***T. jolithus*** **unigene dataset.**
(DOC)

Table S5 **Single-nucleotide polymorphisms (SNP) detected using SOAPsnp after unigene sequence assembly.**
(XLS)

Table S6 **Enzymes encoded in the** ***T. jolithus*** **transcriptome involved in carbon fixation in photosynthetic organisms.**
(XLS)

Table S7 **Enzymes involved in carotenoid biosynthesis of** ***T. jolithus*** **transcriptome.**
(XLS)

Table S8 **Enzymes involved in fatty acid biosynthesis of** ***T. jolithus*** **transcriptome.**
(XLS)
