# Supplementary figures and images for: De Novo Transcriptome Analysis of an Aerial Microalga Trentepohlia jolithus: Pathway Description and Gene Discovery for Carbon Fixation and Carotenoid Biosynthesis
Source: PLoS One. 2014 Sep 25;9(9):e108488. doi: 10.1371/journal.pone.0108488 (PMC4177907; doi:10.1371/journal.pone.0108488)

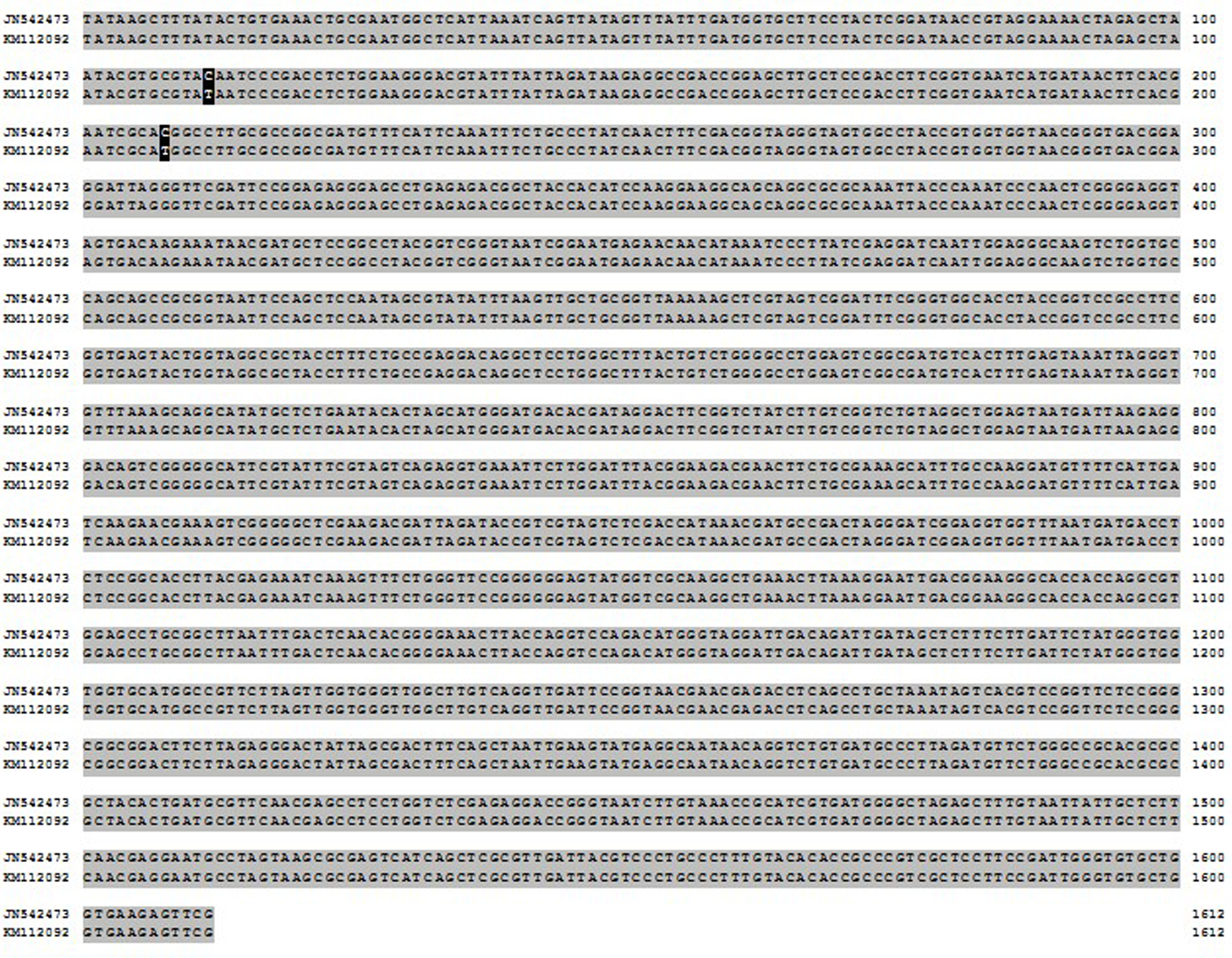

Supplement: Figure S1 — Alignment of 18S ribosomal DNA sequence of T. jolithus (KM112092) with Trentepohlia jolithus var. yajiagengensis var. nov (JN542473). Identical bases were shaded in light grey, and different bases were shaded in black. (TIF) [file pone.0108488.s001.tif]
