# Supplementary material for: De Novo Transcriptome Analysis of an Aerial Microalga Trentepohlia jolithus: Pathway Description and Gene Discovery for Carbon Fixation and Carotenoid Biosynthesis
Source: PLoS One. 2014 Sep 25;9(9):e108488. doi: 10.1371/journal.pone.0108488 (PMC4177907; doi:10.1371/journal.pone.0108488)
Supplement: Table S4 — Summary of the number of repeat units identified from the T. jolithus unigene dataset. (DOC) [file pone.0108488.s005.doc]

**Table S4** **Summary of the number of repeat units identified from the *T. jolithus* unigene dataset**

| **Number of repeats** | **Mono-nucleotide repeats** | **Di-nucleotide repeats** | **Tri-nucleotide repeats** | **Quad-nucleotide repeats** | **Penta-nucleotide repeats** | **Hxta-nucleotide repeats** |
| --- | --- | --- | --- | --- | --- | --- |
| 4 | 0 | 0 | 0 | 0 | 82 | 173 |
| 5 | 0 | 0 | 1,023 | 222 | 2 | 11 |
| 6 | 0 | 772 | 501 | 19 | 0 | 11 |
| 7 | 0 | 452 | 415 | 0 | 2 | 3 |
| 8 | 0 | 352 | 24 | 0 | 0 | 2 |
| 9 | 0 | 429 | 1 | 0 | 1 | 3 |
| 10 | 0 | 355 | 2 | 0 | 0 | 5 |
| 11 | 0 | 164 | 0 | 0 | 0 | 1 |
| 12 | 286 | 16 | 0 | 0 | 0 | 0 |
| 13 | 138 | 0 | 0 | 0 | 0 | 0 |
| 14 | 110 | 0 | 0 | 0 | 0 | 0 |
| 15 | 46 | 0 | 1 | 2 | 0 | 1 |
| 16 | 30 | 0 | 0 | 0 | 0 | 1 |
| 17 | 16 | 0 | 1 | 0 | 0 | 0 |
| 18 | 32 | 0 | 0 | 0 | 0 | 0 |
| 19 | 18 | 0 | 0 | 0 | 0 | 0 |
| 20 | 11 | 0 | 0 | 0 | 0 | 0 |
| 21 | 24 | 0 | 0 | 0 | 0 | 0 |
| 22 | 21 | 0 | 0 | 0 | 0 | 0 |
| 23 | 14 | 0 | 0 | 0 | 0 | 0 |
| 24 | 2 | 0 | 0 | 0 | 0 | 0 |
| 27 | 0 | 1 | 0 | 0 | 0 | 0 |
| SubTotal | 748 | 2,541 | 1,968 | 243 | 87 | 211 |
